# A Case of Stevens–Johnson Syndrome Complicated with Multimatrix System Mesalamine in Ulcerative Colitis

**DOI:** 10.3390/medicina58020276

**Published:** 2022-02-11

**Authors:** Mimari Kanazawa, Keiichi Tominaga, Akira Kanamori, Takanao Tanaka, Satoshi Masuyama, Shoko Watanabe, Keiichiro Abe, Akira Yamamiya, Kenichi Goda, Atsushi Irisawa

**Affiliations:** Department of Gastroenterology, Dokkyo Medical University, 880 Kitakobayashi, Mibu 321-0293, Japan; mimari77@dokkyomed.ac.jp (M.K.); k-akira@dokkyomed.ac.jp (A.K.); tana1986@dokkyomed.ac.jp (T.T.); masu-526@dokkyomed.ac.jp (S.M.); shoko-t@dokkyomed.ac.jp (S.W.); abe9841@dokkyomed.ac.jp (K.A.); akira-y@dokkyomed.ac.jp (A.Y.); goda@dokkyomed.ac.jp (K.G.); irisawa@dokkyomed.ac.jp (A.I.)

**Keywords:** ulcerative colitis, Stevens–Johnson syndrome, multimatrix system mesalamine, severe adverse event

## Abstract

A 41-year-old man was treated with prednisolone (PSL) and multimatrix (MMX) mesalamine for remission induction therapy of ulcerative colitis. PSL was tapered due to successful remission induction treatment. During the treatment course, ocular foreign body sensation, eyelid swelling, ocular conjunctiva hyperemia, facial redness and swelling, watery nasal discharge, stomatitis, anal pain, and reddish puffiness on the bilateral dorsum of the hands appeared, and he was diagnosed with Stevens–Johnson syndrome (SJS). SJS was improved by PSL treatment and intravenous immunoglobulin. MMX mesalamine was the causative agent by drug-induced lymphocyte stimulation test. This is the first reported case of SJS with MMX mesalamine.

## 1. Introduction

The use of 5-aminosalicylic acid (5-ASA) as a basic treatment for ulcerative colitis (UC) has few side effects, and is a drug frequently used for induction and maintenance of remission. In recent years, a sustained-release tablet containing mesalamine (MMX mesalamine) using the multimatrix (MMX) delivery system has been widely used for UC. Currently, salazosulfapyridine (SASP) and three different forms of mesalamine are used in Japan.

Stevens–Johnson syndrome (SJS) and toxic epidermal necrolysis (TEN) are rare, but potentially life-threatening and severe disorders, diseases characterized by widespread epidermal necrosis, and are predominantly medication induced. Although there have been several reports of SJS and TEN caused by mesalamine and SASP, there have been no such reports with MMX mesalamine. Although 5-ASA is widely used as a basic therapeutic agent for UC without immune modulation, it is important to recognize that, like other drugs, it can cause serious side effects, such as SJS and TEN.

## 2. Presentation of Case Report

A 41-year-old man visited his local doctor in March 2020 due to abdominal pain, diarrhea, and bloody stools that started in February 2020. He was diagnosed with UC (total colitis) by colonoscopy and was soon referred to our hospital for treatment. He had no previous medical history and was not taking any medication. He did not consume alcohol and had been smoking 10 cigarettes/day for 21 years since the age of 20.

**Figure 1:**
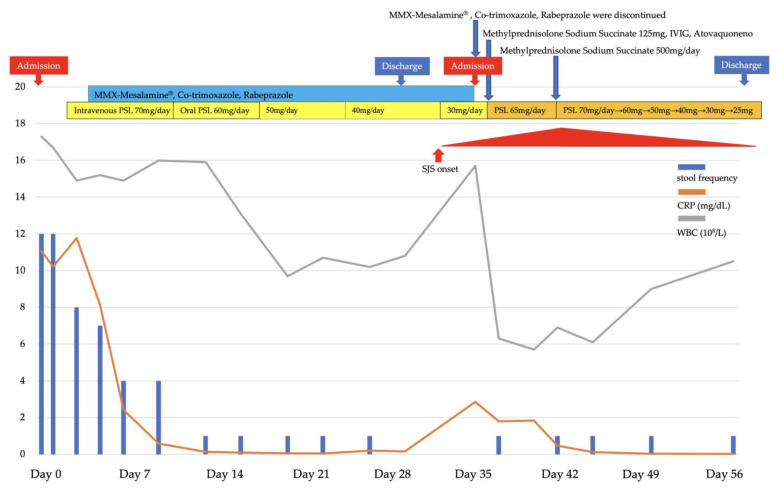
Clinical course of the patient’s condition.

**Figure 2:**
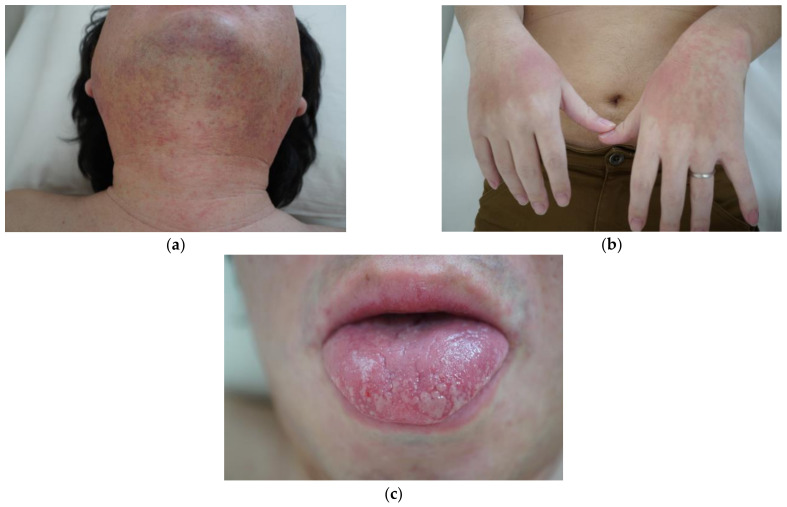
Photos were taken on 15 April 2020. (**a**) A reddish-purple rash is seen on the neck; (**b**) erythematous rash on the dorsum of both hands; (**c**) redness and blistering of the tongue.

The severity of UC at the time of his visit was Lichtiger index of 13 (≥12: severe) [1]. The endoscopy showed a Mayo endoscopic subscore 3 in the entire colon. He was admitted for remission induction of UC and started remission induction therapy with intravenous prednisolone (PSL) 70 mg/day (1 mg/kg/day) and oral MMX mesalamine 4800 mg/day. Oral co-trimoxazole to prevent the development of pneumocystis pneumonia due to immunosuppression associated with high-dose PSL administration, and oral rabeprazole 10 mg/day to prevent gastrointestinal mucosal damage were also started. The UC symptoms improved in about 12 days, and PSL was tapered by 10 mg every week. He achieved remission, had a Lichtiger index of 2, and discharged. MMX mesalamine 4800 mg/day, co-trimoxazole, and rabeprazole 10 mg/day were continued. During the treatment course (Figure 1), PSL was tapered to 30 mg/day, and about 30 days after starting MMX mesalamine, he exhibited eyelid swelling, ocular conjunctiva hyperemia, facial redness and swelling, watery nasal discharge, stomatitis, anal pain, and erythematous swelling of the bilateral dorsum of the hands (Figure 2a–c). We suspected drug-related SJS, or viral infections associated with immunosuppression due to high-dose PSL administration.

**Figure 3:**
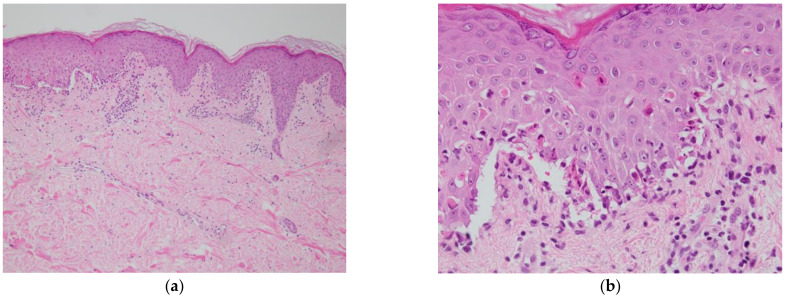
A skin biopsy showing interface dermatitis, and liquid degeneration at the epidermal-dermal border and cleft formation was prominent. There were necrotic keratinocytes in the epidermis and lymphocytic infiltration into the epidermis and around subepidermal microvessels. (**a**) Hematoxylin and eosin staining (100×); (**b**) hematoxylin and eosin staining (400×).

Nikolsky’s sign (a phenomenon in which the epidermis of apparently normal skin becomes detached and erosive when mild pressure is applied) was positive. The ocular lesions included extensive corneal erosion and severe conjunctival hyperemia. Laboratory tests showed mild liver dysfunction with AST 31 U/L and ALT 65 U/L. WBC was 15.7 × 103/μL and CRP was 2.85 mg/dL, and anemia was present with Hb 11.2 g/dL. Eosinophil was 0.8% and IgE was 12.6 IU/L. Although SJS may occur with mycoplasma and some viral infections, bacterial and viral infections were ruled out as the patient was HSV-IgG positive, mycoplasma negative, ASO negative, and CMVAg negative (Table 1). The skin biopsy showed interface dermatitis, and liquid degeneration at the epidermal–dermal border and cleft formation was prominent. There were necrotic keratinocytes in the epidermis and lymphocytic infiltration into the epidermis and around subepidermal microvessels (Figure 3a,b). We considered the patient’s symptoms to be drug-induced SJS and discontinued all three suspected drugs, which were MMX mesalamine, co-trimoxazole and rabeprazole sodium, and started intravenous methylprednisolone 125 mg/day and intravenous immunoglobulin. Before the results of the drug-induced lymphocyte stimulation test (DLST) became clear, we also started atovaquone instead of co-trimoxazole to prevent the onset of pneumocystis pneumonia due to immunosuppression associated with high doses of PSL, and famotidine instead of rabeprazole to prevent gastrointestinal mucosal damage. In the DLST that was submitted prior to the start of treatment to identify the causative drug, MMX mesalamine was positive, co-trimoxazole and rabeprazole negative, and MMX mesalamine was determined to be the causative agent.

**Figure 4:**
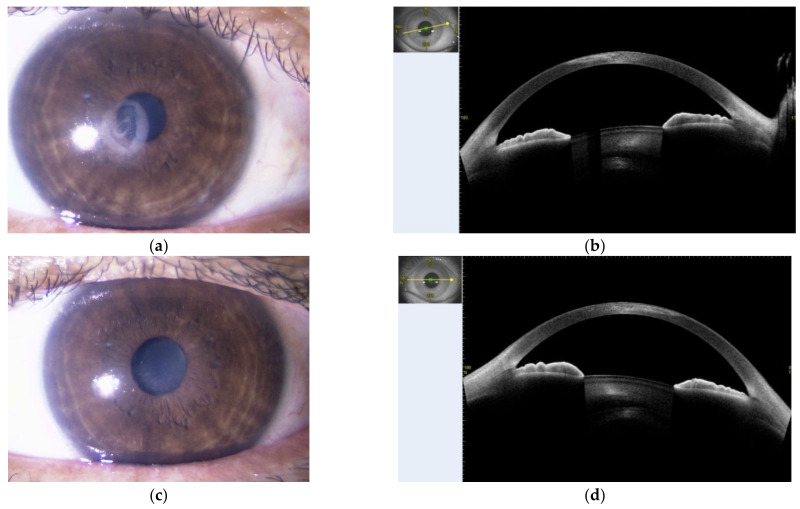
Photos were taken on 29 July 2020. In the bilateral eye, there is opacity extending to the corneal parenchyma. (**a**) Right eye ball; (**b**) right eye ball’s optical coherence tomography; (**c**) left eye ball; (**d**) left eye ball’s optical coherence tomography.

The symptoms continued to worsen even after the start of treatment, with widely distributed erythema, blistering, and epidermal peeling over the entire body, including the palms and soles, and mucosal lesions in the transition area between the lips and the oral and anal mucosa. In addition, ocular pain increased and corneal opacity and pseudomembrane formation were observed, and consequently methylprednisolone 500 mg/day was administered intravenously for 3 days, and symptoms began to improve. Thereafter, we gradually tapered PSL from 70 mg/day. The skin and mucosal lesions showed a tendency to heal, and some nails were deformed and dropped off, but later recovered. As for the ocular lesions, visual impairment due to opacity of the corneal parenchyma remained as a sequela (Figure 4a–d). Fortunately, no exacerbation of UC was observed during this course. Since MMX causes SJS, it was decided that a 5-ASA formulation could not be used and that an immunomodulator would be used to maintain UC remission.

## 3. Discussion

This is a case of UC with SJS caused by MMX mesalamine. SJS and TEN develop 1–3 weeks after the initiation of the causative agent [2,3]. Based on international standards, SJS and TEN are distinguished by their percentage of body surface area, with SJS being less than 10%, overlap syndrome being 10–30%, and TEN being more than 30% [4]. The fatality rate can be high, ranging from 3–5% in SJS and 20–30% in TEN [5]. In addition, about 50–89% of SJS/TEN patients develop ocular complications, which may lead to serious adverse events such as corneal damage and vision loss [6,7,8]. In 1987, Roujeau et al. highlighted the significance of polymorphisms in human leukocyte antigen (HLA) in the pathogenesis of SJS/TEN [9]. The HLA-B*44:03 found in the present case has been reported to be associated with SJS/TEN with severe ocular complications related to common cold medications [10,11].

In general, DLST has low sensitivity and high specificity in drugs such as antituberculosis drugs and rheumatic drugs. Saito et al. reported that the sensitivity and specificity of DLST for mesalamine were 0.240 and 0.805, respectively [12]. As with other drugs, DLST for mesalamine has low sensitivity and high specificity, so a positive DLST is strongly suspected to be caused by the drug. Therefore, DLST may be useful in the definitive diagnosis of the causative agent of SJS and TEN. In this case, DLST of MMX mesalamine was positive, while co-trimoxazole and rabeprazole were negative. Based on the sensitivity and specificity, we thought that co-trimoxazole and rabeprazole were unlikely to be involved in the development of SJS in this case, and came to the diagnosis of SJS by MMX mesalamine.

The basic drug 5-ASA that can treat UC without immunosuppression, has few side effects, and is used from remission induction to long term remission maintenance. Several mesalamines with different drug delivery systems and SASP have been used to improve delivery to the colon, the main site of disease in UC. Among these, there have been several reports of SJS and TEN occurring when using mesalamine and SASP [13,14,15,16,17,18,19,20,21], but there have been no similar reports when using MMX mesalamine (Table 2).

The mechanism of drug release obtained with the MMX system avoids the release of the embedded mesalamine until the tablet is exposed to pH 7 or higher, which is normally reached in the terminal ileum. After reaching the terminal ileum, the activity of the tablet core, which is composed of hydrophilic excipients (thought to drive the tablet to swell into a viscous gel mass, and slowing the release of the drug) and lipophilic excipients (thought to slow the penetration of aqueous fluids into the tablet core), results in a homogenous and prolonged exposure of the entire colonic mucosa to the embedded mesalamine [22]. MMX mesalamine is capable of sustained release of mesalamine throughout the colon, which differentiates it from other 5-ASA formulations. In addition to mesalamine, the MMX system has been implemented in budesonide, parnaparin, rifampicin, and other drugs, and has been used or is being considered for use in clinical practice. All of the aforementioned drugs have been reported to have a high safety profile and have not been associated with serious adverse events [23,24,25,26,27,28]. We speculated that the MMX system, with its slow release of drugs, may reduce the incidence of SJS and TEN.

In particular, this patient was receiving a high dose of PSL for remission induction of UC at about the same time as the causative agent. Although the pathogenesis of SJS is unknown, it is thought that cytotoxic T cells activated by drugs taken into the body may directly induce apoptosis of epidermal cells, or cytokines (e.g., TNF-α) produced by activated cytotoxic T cells may indirectly cause cytotoxicity [29,30]. It has been shown that the dose of PSL is related to the function of cellular immunity [31,32] and it is possible that in the present case, the onset of SJS was suppressed by the use of high-dose PSL, and the symptoms only became apparent as PSL was gradually tapered. Furthermore, since this patient was receiving a relatively high dose of PSL at the time of onset, it is possible that SJS-induced fever could have been masked.

Lastly, based on previous reports, cases in which PSL was administered at the same time as the causative drug tended to have a longer time to onset than cases in which PSL was not administered Lastly, based on previous reports, cases in which PSL was administered at the same time as the causative drug tended to have a longer time to onset than cases in which PSL was not administered (Table 2).

## 4. Conclusions

A relatively safe drug with a low incidence of side effects, 5-ASA is widely used as a basic treatment for UC. However, it is important to recognize that it can cause serious side effects such as SJS and TEN in susceptible individuals. Although the incidence of SJS and TEN is extremely low, they have a relatively high fatality rate and can leave serious sequelae, therefore, prompt therapeutic intervention is critical when diagnosed.

## Figures and Tables

**Table 1:** Laboratory data on admission.

		Normal Range			Normal Range
AST	31 U/L	13–30	WBC	1.57 × 10^3^/μL	3.30–8.60
ALT	65 U/L	10–42	RBC	3.91 × 10^6^/μL	4.35–5.55
ALP	254 U/L	106–322	Hb	11.2 g/dL	13.7–16.8
LD	155 U/L	124–222	Plt	385 × 10^9^/L	158–348
γGTP	130 U/L	13–64	ESR (1 hr)	40 mm	2–10
T-Bil	0.4 mg/dL	0.4–1.5	ferritin	38.4 ng/mL	21.8–274.6
UN	16 mg/dL	8–20	IgG	752 mg/dL	870–1700
Cre	0.59 mg/dL	0.65–1.07	IgE	12.6 IU/mL	<170
Alb	3.0 mg/dL	4.1–5.1	HSV-IgG	35.6 (+)	<2.0
Na	139 mmol/L	138–145	CMVAg	negative	
K	4.4 mmol/L	3.6–4.8	Mycoplasma	negative	
Cl	104 mmol/L	101–108	ASO	negative	
CRP	2.85 mg/dL	<0.14	TARC	215 pg/mL	<450

Abbreviations: AST: aspartate aminotransferase; ALT: alanine aminotransferase; ALP: alkaline phosphatase, LDH: lactate dehydrogenase; γGTP: γ-glutamyl transpeptidase; T-Bil: total bilirubin; UN: urea nitrogen; Cre: creatinine; Alb: albumin; Na: sodium; K: potassium; Cl: chloride; CRP: C-reactive protein; WBC: white blood cell; RBC: red blood cell; Hb: hemoglobin; Plt: platelet; ESR: erythrocyte sedimentation rate; IgG: immunoglobulin G; IgE: immunoglobulin E; HSV: *Herpes simplex virus;* CMVAg: cytomegalovirus antigenemia; ASO: anti-streptolysin O; TARC: thymus and activation-regulated chemokine.

**Table 2:** Reported cases of Mesalamine and Salazosulfapyridine related Stevens–Johnson syndrome/toxic epidermal necrolysis.

No.	Author	Published	Age	Sex	Disease	Diagnosis	Causative Drug	Time to Onset of SJS	Nikolsky’s Sign	Treatment	Outcome
1	Maddocks JL	1980	39	M	UC	TEN	SASP	60 days	-	intravenous methylprednisolone	death
2	Tolia V	1992	17	M	UC	SJS	SASP	-	-	-	improved
3	Tolia V	1992	13	F	UC	SJS	SASP	-	-	-	improved
4	Tolia V	1992	16	M	UC	SJS	SASP	-	-	-	improved
5	Lemoli E	2006	-	M	UC	TEN	mesalamine	-	-	steroids, antimycotics, antibiotics	improved
6	Fukunaga K	2007	17	M	UC	TEN	mesalamine	19–34 days	positive	intravenous methylprednisolone	improved
7	Tremblay L	2011	36	F	UC	SJS	SASP	11 days	positive	symptomatic treatment	improved
8	Tremblay L	2011	19	F	UC	SJS	SASP	21 days	-	symptomatic treatment	improved
9	Zizi N	2015	33	F	-	SJS/ TEN	SASP	15 days	positive	symptomatic treatment	improved
10	Núñez Ortiz A	2018	46	F	UC	SJS	mesalamine	14 days	negative	intravenous corticosteroids	improved
11	Xiong H	2018	61	F	UC	SJS	SASP	12 days	-	-	-
12	Viola A.	2019	32	F	CD	SJS	SASP	within 30 days	-	intravenous steroid, antihistamine	improved
Present case			41	M	UC	SJS	MMX mesalamine	28 days	positive	intravenous steroid, IVIG	improved

Abbreviations; M: male, F: female, UC: ulcerative colitis, CD: Crohn’s disease, TEN: toxic epidermal necrolysis, SJS: Stevens–Johnson syndrome, SASP: salazosulfapyridine, MMX: multimatrix, IVIG: intravenous immunoglobulin.

## Data Availability

Not applicable.
